# Supplementary material for: A General and Modular Approach to Solid-State Integration of Zero-Dimensional Quantum Systems
Source: Nano Lett. 2025 Sep 3;25(37):13787–94. doi: 10.1021/acs.nanolett.5c03125 (PMC12447553; doi:10.1021/acs.nanolett.5c03125)
Supplement: Supplementary file 1 [file nl5c03125_si_001.pdf]

Supporting Information

A general and modular approach to solid-state integration of zero-dimensional quantum systems

Marzieh Kavand[1,6], Zoe Phillips[1†], William H. Koll[1†], Morgan Hamilton[1†], Ethel Perez-Hoyos[1], Rianna Greer[2], Ferdous Ara[1], Daniel Pharis[1], Kian Maleki[3], Mingyu Xu[4], Takashi Taniguchi[5], Paul Canfield[4], Michael E. Flatté[3,7], Danna E. Freedman[2], Jay Gupta[1], Ezekiel Johnston-Halperin[1*]

[1]Department of Physics, The Ohio State University, Columbus, Ohio 43210, USA.
[2]Department of Chemistry, Massachusetts Institute of Technology, Cambridge, Massachusetts 02139, USA.
[3]Department of Physics and Astronomy, University of Iowa, Iowa City, Iowa, USA.
[4]Ames National Laboratory and Department of Physics and Astronomy, Iowa State University, Ames, Iowa, 50010, USA.
[5]Research Center for Materials Nanoarchitectonics, National Institute for Materials Science, 1-1 Namiki, Tsukuba 305-0044, Japan.
[6]Department of Physics and Astronomy, The University of Alabama, Tuscaloosa, Alabama, 35487, USA.
[7]Department of Applied Physics and Science Education, Eindhoven University of Technology, 6500 MB Eindhoven, The Netherlands

[†]These authors contributed equally to this work.
[*]Correspondence to: johnston-halperin.1@osu.edu

Supplementary 1: Stacking and fabrication of hBN tunnel junction (hBN-TJ) devices

After exfoliation of graphite on the Si/SiO_2 substrate, we identify the top and bottom MLG flakes with appropriate thickness and shapes using an optical microscope. To create a stack of the heterostructure with 2D materials, we initiate the process by transferring the top MLG. This is accomplished by picking it up using a polycarbonate (PC) film coated on a polydimethylsiloxane (PDMS) stamp and a glass slide at a temperature of 90°C. Subsequently, hBN is transferred onto the top MLG flake, also picked up at a temperature of 90°C. This procedure ensures that the interfaces of the hBN layer remain clean, with no direct contact with solvents or polymers. In the final step, the top MLG/hBN layers are delicately placed onto the bottom MLG, previously exfoliated on a Si/SiO2 substrate, at a temperature of 105°C. The entire stack is then heated to 150°C for about 10 minutes for the adhesion of the 2D heterostructure onto the substrate. After allowing the stack to cool, we dissolve the polycarbonate (PC) film by immersing the stack in chloroform for approximately 4 minutes, adjusting the duration based on the amount of PC film present on the substrate. The subsequent step involves annealing the sample for a duration of 90 minutes at a temperature of 350°C under vacuum conditions. Specifically, we employ a gradual thermal ramp-up and ramp-down, with a 90-minute rise from room temperature to 350°C, 90

minutes of annealing at 350°C, followed by a 90-minute descent to room temperature to prevent thermal shock. This process eliminates any residues of polymers and solvents from the stack while enhancing the interfacial bonding between the layers of the stack. We employ electron beam lithography to pattern gold contacts for establishing electrical connections. Following the spin coating of MMA and PMMA (both deposited at 3000 RPM for 45 seconds), we use electron beam to write the contact patterns. The pattern is then developed with a mixture of MIBK/IPA for approximately 70 seconds, adjusting the duration based on the amount of resist residue on the sample. Finally, a 5 nm Chromium (Cr) film is deposited, followed by a 150 nm layer of gold (Au) for the metal contacts. We utilize acetone for the lift-off process of the gold contacts. At the end the sample is wire-bonded on a chip carrier for transport measurements.

Supplementary 2: Atomic force microscopy (AFM) imaging and tunneling spectroscopy of hBN tunnel junctions with varying hBN thickness

Figure S1 shows the AFM image of the three-layer stack (MLG/hBN/MLG) device, with the optical image shown in Figure 1b in the main text. From the AFM data analysis, the thickness of the hBN tunnel barrier is estimated to be 3.01 ± 0.66 nm and an active area of the device is $3.1\ \mu m^2$. Figure S2 shows dI/dV measurements for three hBN-TJ devices with various commercial hBN flack thicknesses: 2.4, 2.7 and 4.7 nm. The data reveal that increasing the hBN thickness led to a decrease in direct tunneling and resonance peaks become broader with less sharp resonance features.

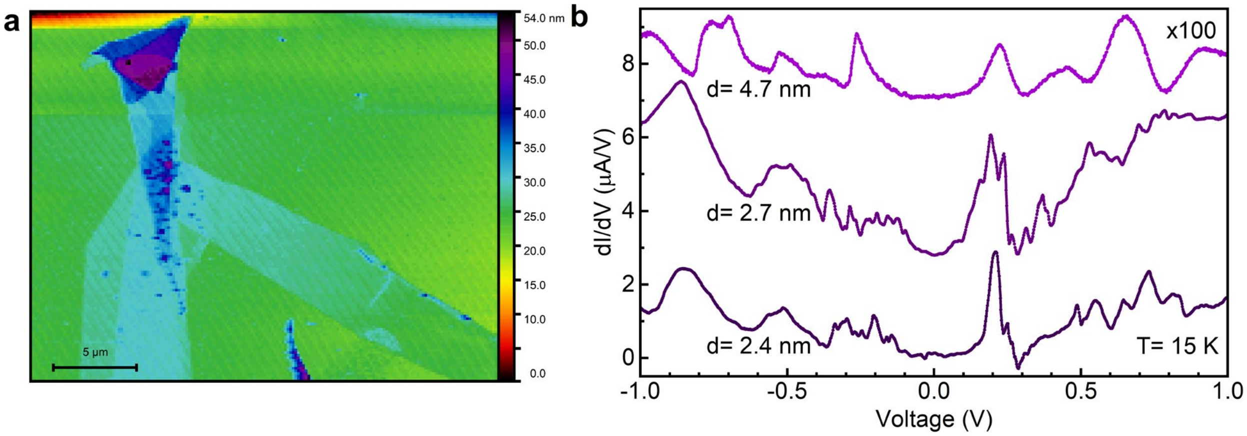

Figure S1. AFM image and the dependency of tunneling spectroscopy on hBN thickness. (a) An AFM scan image of the three-layer stack (MLG/hBN/MLG) for the device, with the corresponding optical image shown in Figure 1b. (b) dI/dV measurements for three hBN-TJ devices with various commercial hBN flack thicknesses.

Supplementary 3: Tunneling spectroscopy of the tunnel junction device with MANA hBN

The MANA hBN-TJ device discussed in Figure 2a of the main text does not exhibit substantial resonant tunneling overall. Figure S2 provides a zoomed-in view of the data from Figure 2a, focusing on the range from -0.4 V to +0.4 V. A small and broad resonance is observed around -0.2 V.

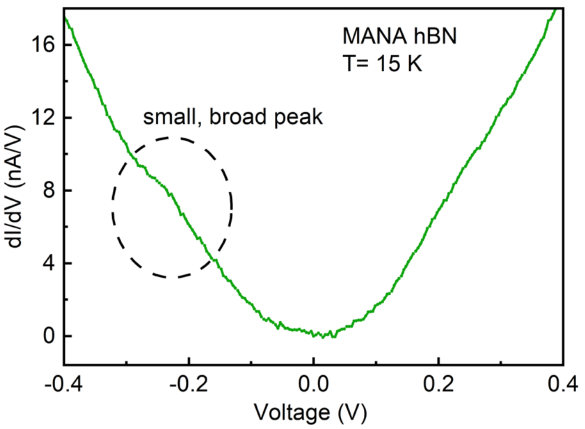

Figure S2. Significant suppression of resonance tunneling in MANA hBN. Zooming in on the dI/dV data in Figure 2a (green curve) shows only a small and broad resonant tunneling peak around -0.25 V for the MANA hBN-TJ device.

Supplementary 4: Ames hBN synthesis

Synthesis of hBN takes place in a high-pressure furnace at 3.2 GPa, in a process that co-synthesizes both hBN and MgB_2. The temperature is increased to 1380 °C before slowly cooling down to 650 °C. The flux ratio of Mg:B elements in a BN crucible is 1:0.7. The synthesis process for carbon-dopped hBN is similar to that described above with the exception that 2% of the B is replaced by C, resulting in a flux ratio of Mg: B: C of 1: 0.686: 0.014.

Supplementary 5: Effect of temperature on tunneling spectroscopy in a C-doped hBN tunnel junction device

Figure S3a shows the tunneling spectroscopy of the C-doped hBN tunnel junction device discussed in Figure 3b of the main text at various temperatures: 15 K, 80 K, 100 K, 150 K, and 290 K. The data reveals that the resonant tunneling becomes broader as the temperature increases, and above 80 K, the line shapes become distorted. As shown in Figure S3b, for the sharp peak around -0.94 V, the line shapes were fitted with linear functions (to remove the baseline) and Lorentzian functions, normalized by their maximum for temperatures ranging from 15 K to 80 K. Above 80 K, the line shapes are distorted and cannot be fitted. Figure S3c shows the fitting results of a quadratic-sum model ($FWHM(T) = \sqrt{\Delta^2 + (\alpha T)^2}$ where Δ and αT represent intrinsic and thermal broadening, respectively) to the full width at half maximum (FWHM) values obtained from Figure S3b. The blue curve represents the quadratic-sum fit to the data. The fitted values are $\Delta = 8.8 \pm 0.3\ \mathrm{mV}$ and $\alpha T = 0.12 \pm 0.006$. The fitted values underestimate the zero-temperature

inhomogeneous broadening, indicating the presence of additional broadening mechanisms beyond those captured by the simple quadratic-sum model. To account for artifact broadening due to lock-in modulation, we performed careful measurements to verify that the modulation amplitude does not affect the line shape. Figure S4 shows the dI/dV spectra for three different modulation amplitudes: 2.5, 5.0, and 7.5 mV. We used a modulation amplitude of 5.0 mV for recording the temperature dependence data in Figure S3, as the line shape remained unchanged compared to that at the lower amplitude of 2.5 mV.

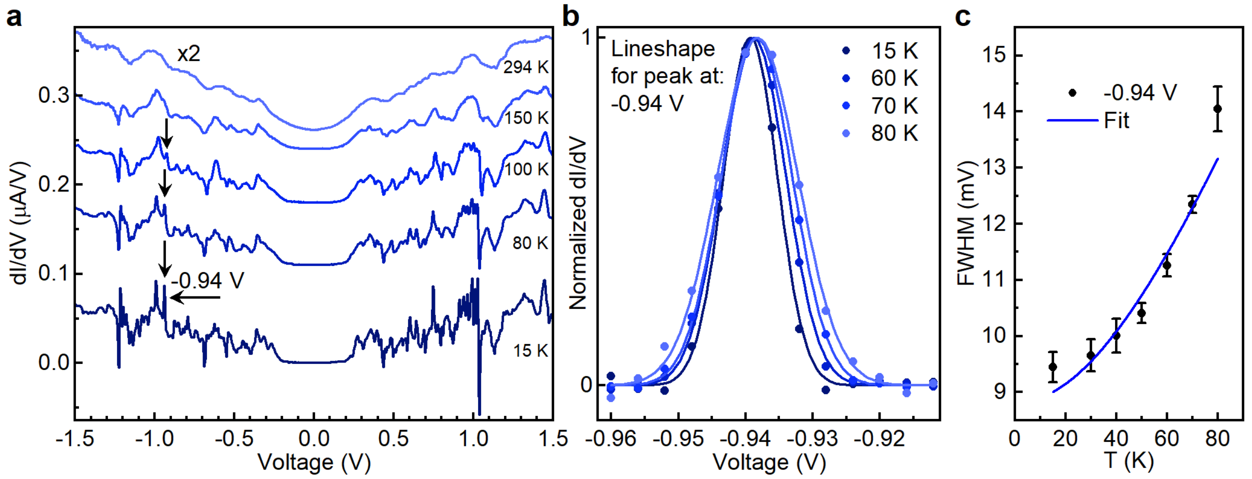

Figure S3. Temperature dependency of tunneling spectroscopy for a C-doped hBN tunnel junction device. (a) dI/dV spectra at various temperatures: 15 K, 80 K, 100 K, 150 K, and 290 K. (b) Line-shape analysis of the sharp resonant peak at -0.94 V. The blue lines are the fit results. (c) The full width at half maximum (FWHM) of the line-shape fitted results as a function of the temperature.

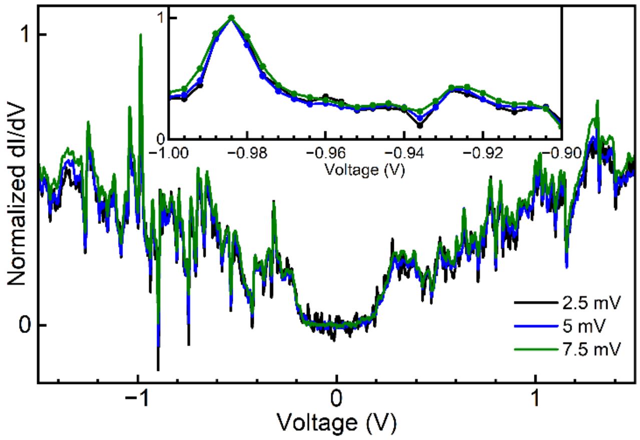

Figure S4. Normalized dI/dV spectra measured with three different lock-in modulation amplitudes: 2.5 mV, 5.0 mV, and 7.5 mV.

Supplementary 6: VOPc synthesis and characterization

a) General considerations: Vanadyl (IV) sulfate pentahydrate was purchased from Alfa Aesar, 1-pentanol and phthalonitrile were purchased from Sigma-Aldrich, and diazabicyclo [5.4.0] undec-7-ene (DBU) was purchased from Chem-Impex International. All chemicals were used as received.

a) Synthesis: Vanadyl (IV) phthalocyanine (VOPc) was prepared according to an adapted literature procedure[1]. A 250 mL round-bottom flask was charged with vanadyl (IV) sulfate pentahydrate (0.7604 g, 3 mmol, 1.2 eq.), phthalonitrile (1.2817 g, 10 mmol, 4 eq.), DBU (1.5 mL, 10 mmol, 4 eq.), 40 mL 1-pentanol, and a stir bar. The flask was then refluxed at 145 °C for 16 hours. The dark blue-purple product was isolated via vacuum filtration, rinsed with 100 mL deionized water, 100 mL ethanol, and 500 mL ethyl acetate, and dried for 20 minutes. Product yield was 0.2998 g (20.7%). MALDI-TOF mass spectrometry (m/z): [M]+ calculated for C32H16N8VO, 579.089, 580.092, 581.095, 580.086; found 578.859, 579.037, 579.140, 579.919. FTIR (cm-1): 436.9, 504.9, 567.8, 637.9, 723.4, 750.2, 774.9, 800.7, 836.8, 872.8, 896.5, 958.4, 998.5, 1072.7, 1117.1, 1156.2, 1285.0, 1330.4, 1413.8, 1460.2, 1496.3, 1606.5, 3668.5, 3744.8, 3848.9.

b) Powder X-Ray diffraction: Bulk purity was evaluated through powder X-ray diffraction (PXRD) (Figure S5). A powdery sample of VOPc was loaded into a PXRD mask between pieces of Kapton tape and PXRD patterns were collected on a STOE STADI MP diffractometer equipped with CuKα1 radiation (λ = 1.5406 Å) located at the Integrated Molecular Structure Education and Research Center of Northwestern University. Simulation of PXRD pattern was produced from crystallographic data previously collected[2].

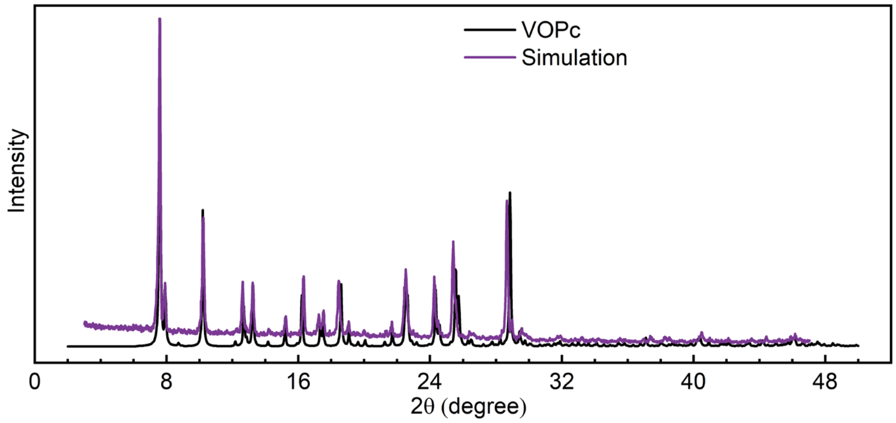

Figure S5. Powder X-ray diffraction (PXRD) patterns for VOPc. simulated (black) vs. experimental (purple) Data were collected at room temperature using CuKα1 radiation (λ = 1.5406 Å). Simulation of PXRD pattern was based on single crystal X-ray diffraction data of VOPc as reported in Reference 2.

c) Other physical measurements: MALDI-TOF mass spectrometry (MS) measurements were collected on a Bruker RapiFlex Tissue Typer mass spectrometer located at the Integrated Molecular Structure Education and Research Center of Northwestern University. No matrix was necessary for the ablation and desorption of VOPc in MALDI-TOF MS measurements. Infrared spectra were recorded on a Bruker Alpha II Compact FTIR spectrometer equipped with an attenuated total

reflectance accessory located at the Department of Chemistry Instrumentation Facility of Massachusetts Institute of Technology.

Supplementary 7: Scanning tunneling microscopy (STM) and spectroscopy (STS) of VOPc on a hBN/HOPG half device.

Figures S6a and b show defects with atomic resolution in STM topographic images of a bare monolayer of hBN on HOPG. The atomic defects are sources of variation in the tunneling spectroscopy (STS) in the half hBN device shown in Figure 3d in the main text. In addition, in Figure S6b there is a moire pattern (period ~14nm) because of the lattice mismatch of the hBN and the HOPG. Figure S6c shows scan of STS under constant-current (+2.3V, 326pA) for a VOPc bilayer on hBN/HOPG measured simultaneously with the topographic image in Figure 3c in the main text. The long-distance fluctuations in contrast may reflect defects or the moiré lattice in the underlying hBN.

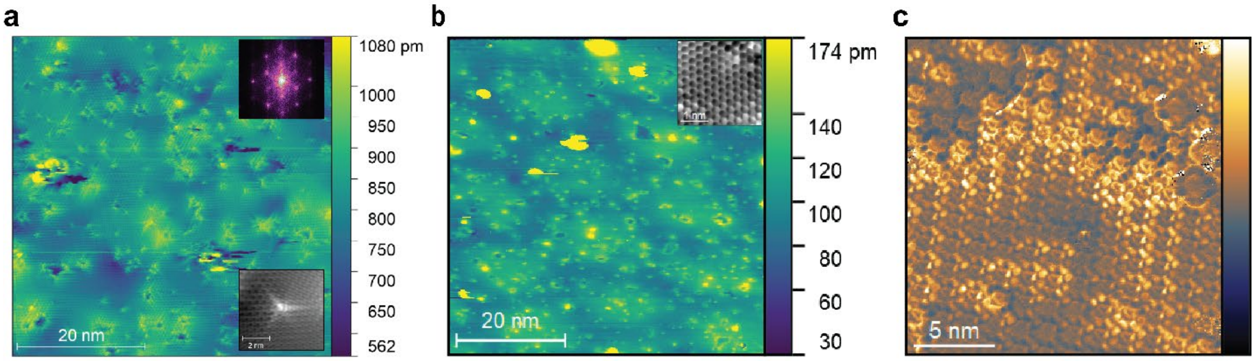

Figure S6. Sources of spatial variation in tunneling spectroscopy of VOPc on a hBN/HOPG half device. (a) STM wide-area topographic image of a bare monolayer hBN on HOPG with atomic resolution and defects. Top inset, 2D-FFT of image, bottom inset, close-up of a single-point defect. (b) Another STM topographic image of a bare monolayer hBN on HOPG. There is a moire pattern (period ~14nm) due to the lattice mismatch of the hBN and the HOPG. Inset, hBN atomic resolution. (c) Constant-current dI/dV map of VOPc bilayer on hBN/HOPG acquired simultaneously with topographic image in Figure 3c. STM imaging conditions: (a) +2.6V, 78pA; (b) +2.37V, 20pA inset, +620mV, 190pA; and (c) +2.3V, 326pA.

Figures S7a-c show a representative dataset of STM constant height images and marked locations at which STS were acquired. Figure S7a shows typical STS data for the following locations: center of molecule in the film, lobe of molecule in the film, center of adsorbed molecule, lobe of adsorbed molecule. The spectrum acquired on the center of the molecule (green) shows multiple pairs of sharp peaks at roughly ± 1 V and ± 2 V. This is consistent with the energies of the HOMO/LUMO pair, and the spin-polarized V states predicted by the pDOS calculations in ref 50. Figure S7b likewise compares spectra measured at the same relative location (center vs lobe) but in different unit cells. Narrow peaks that are present on one unit cell may be absent in another, yet broad spectral features tend to occur at roughly the same energy. In

Figure S7c, the spectrum acquired on the center of the O-up molecule (relatively rare on this surface) exhibits peaks multiple pairs of peaks just like its O-down counterpart in Figure S7a.

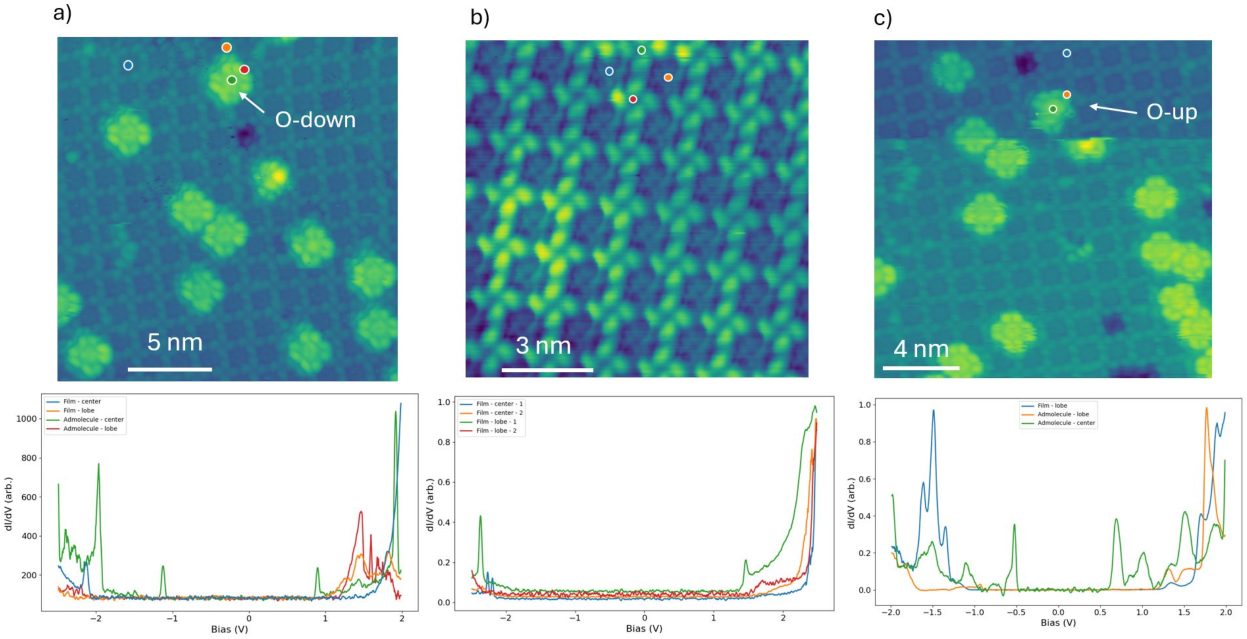

Figure S7. Selected STS on VOPc/hBN surface, with corresponding tip locations. Top row: constant current STM topography of VOPc/hBN "half-stack". Colored dots (blue, red, green, yellow) indicate the location that the tip was marked during acquisition of STS data. Bottom row: STS measurements acquired at various locations in the image directly above. Traces are colored according to tip position. (a) Comparison of spectra on the center/lobe of molecules in the film, and adsorbed atop the film. (b) Spectra revealing the typical variation of the STS signal for nominally identical locations in the unit cell of the film. (c) Spectra acquired in the vicinity of an O-up adsorbed VOPc molecule.

Supplementary 8: Stacking and fabrication of VOPc tunnel junction (VOPc-TJ) device

Heterostructures were assembled in a five-layer stack using a modification of the process described for MLG/hBN/MLG structures above: an initial layer of MANA hBN with target thickness of 1.5 nm was stacked onto a bottom MLG electrode, and then the polycarbonate film was removed using chloroform. This structure was transferred into the STM chamber for deposition of VOPc using an identical protocol to the one reported for STM studies above. The stack, now with VOPc as the top layer, was then removed from the STM chamber and a second MANA hBN layer (target thickness of 3.5 nm) and the top MLG electrode were placed on top with an alignment that prevents shorting of the two MLG layers, resulting in a stack structure of MLG/hBN(1.5 nm)/VOPc bilayer/hBN (3.5 nm)/MLG (see inset to Figure 3e).

Supplementary 9: Direct tunneling and resonant tunneling models

In the direct tunneling model (Equation 1 in the main text), I_b is resonant tunneling current, m_{eff} is the effective mass of electron in hBN[3], 0.588 m_o , where m_o is mass of free electron, q is charge of electron, h is Planck constant, E is energy, and $\mu = 3.77$ eV is chemical potential of graphene[4], and κ is $\pi\sqrt{8\, m_{eff}}/h$. f_0, f_1, and f_2 are three free parameters. The parameter f_0 rescales the current to match the effective area of the sample, and $f_1$is the effective height of the barrier which is generally the sum of the chemical potential and thermionic work function. The parameter f_2 modifies the applied electric field, to an effective F that appears in Equation 1 corresponding to the charge q times the electric field E. Figure S8a-c show the results of the direct tunneling calculations corresponding to the data discussed in Figure 2a. of the main text.

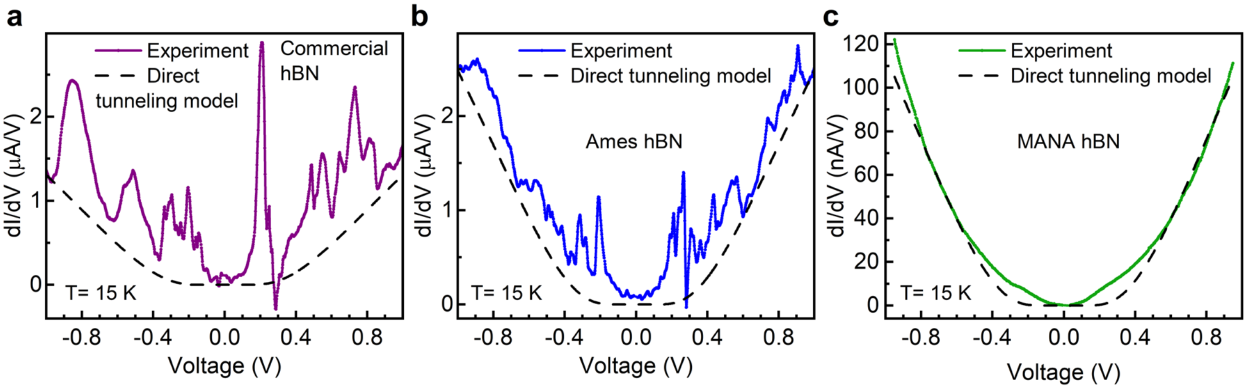

Figure S8. Direct tunneling model. dashed curves are calculated direct tunneling at $T = 15$ K for various hBN-TJ device: (a) commercial hBN, (b) Ames hBN**,** and (c) MANA hBN.

In the resonant tunneling model (Equation 2 in the main text), I_d is resonant tunneling current, $f(E)$ is Fermi-Dirac distribution, and Gaussian function for each resonance is

$$\Gamma(E) = d_0\, e^{-\left(\frac{E-d_1}{d_2}\right)^2} \qquad (S1)$$

where, d_0, d_1, and d_2 are the conductance peak height, voltage of the peak, and linewidth, respectively.

Supporting Information References

1. Tomoda, H.; Saito, S.; Shiraishi, S. Synthesis of Metallophthalocyanines from Phthalonitrile with Strong Organic Bases. *Chem. Lett.* **12**, 313–316 (1983).
2. Ramadan, A. J.; Rochford, L. A.; Keeble, D. S.; Sullivan, P.; Ryan, M. P.; Jones, T. S.; Heutz, S. Exploring High Temperature Templating in Non-Planar Phthalocyanine/Copper Iodide (111) *Bilayers. J. Mater. Chem. C* **3**, 461–465 (2014).
3. Y. Malozovsky et al., Accurate Ground State Electronic and Related Properties of Hexagonal Boron Nitride (h-BN), *Journal of Modern Physics* 11, no. 6 (2020).
4. Jannatul Mawwa et al., In-Plane Graphene/Boron Nitride Heterostructures and Their Potential Application as Toxic Gas Sensors, *RSC Advances* 11, no. 52 (2021).
